# The EGFR/miR-338-3p/EYA2 axis controls breast tumor growth and lung metastasis

**DOI:** 10.1038/cddis.2017.325

**Published:** 2017-07-13

**Authors:** Yingchun Liang, Xiaojie Xu, Tao Wang, Ying Li, Wenye You, Jing Fu, Yang Liu, Shuai Jin, Quanbo Ji, Wei Zhao, Qi Song, Ling Li, Tian Hong, Junjian Huang, Zhaohui Lyu, Qinong Ye

**Affiliations:** 1 Department of Medical Molecular Biology, Beijing Institute of Biotechnology, Collaborative Innovation Center for Cancer Medicine, Beijing, China; 2 Department of Oncology, 307 Hospital of People's Liberation Army, Beijing, China; 3 Department of Oncology, PLA General Hospital, Beijing, China; 4 Department of Thoracic Surgery, PLA General Hospital, Beijing, China; 5 Department of Orthopedics, PLA General Hospital, Beijing, China; 6 Department of Oncology, The General Hospital of the PLA Rocket Force, Beijing, China; 7 Department of Endocrinology, PLA General Hospital, Beijing, China

## Abstract

Dysregulation of the epidermal growth factor receptor (EGFR) promotes cancer cell growth, invasion and metastasis. However, its relevant downstream effectors are still limited. Here, we show that EGFR promotes breast tumor growth and metastasis by downregulating the tumor suppressor micoRNA-338-3p (miR-338-3p) and activating the EYA2 (EYA transcriptional coactivator and phosphatase 2) oncoprotein. EGFR represses miR-338-3p expression largely through HIF1*α* transcription factor. miR-338-3p inhibits EYA2 expression by binding to the 3′-untranslated region of EYA2. EGFR increases EYA2 expression via HIF1*α* repression of miR-338-3p. Through the miR-338-3p/EYA2 pathway, EGFR increases breast cancer cell growth, epithelial-to-mesenchymal transition, migration, invasion and lung metastasis *in vitro* and in a allograft tumor mouse model *in vivo*. In breast cancer patients, miR-338-3p expression negatively correlates with the expression of EGFR and EYA2, EGFR status positively associates with EYA2 expression, and miR-338-3p and EYA2 predict breast cancer lung metastasis when expressed in primary breast cancers. These data suggest that the miR-338-3p/EYA2 axis contributes to EGFR-mediated tumor growth and lung metastasis and that miR-338-3p activation or EYA2 inhibition or combination therapy targeting EGFR/miR-338-3p/EYA2 axis may be a promising way to treat patients with metastatic cancer.

The epidermal growth factor receptor (EGFR) is a member of the ErbB (avian erythroblastosis oncogene B) family of receptors and activates multiple signaling pathways, including mitogen-activated protein kinase (MAPK)/extracellular signal-regulated kinases (ERK) and phosphoinositide-3-kinase (PI3K)/V-AKT murine thymoma viral oncogene homolog (AKT) pathways.^1, 2, 3^ EGFR activation regulates many biological processes, such as cell proliferation, invasion, metastasis and apoptosis.^4, 5, 6^ EGFR is overexpressed in various human cancers, including lung cancer, breast cancer, colon cancer and glioblastoma, and is associated with tumor malignancy and poor prognosis.^7, 8, 9, 10^ Thus, EGFR and its downstream signaling effectors have become targets for cancer therapy.^11^

Approximately 90% of deaths associated with cancer are due to distant metastases.^12^ Many cancers can metastasize anywhere in body but primarily metastasizes to some organs or tissues. For instance, lungs and bones are frequent sites of breast cancer metastasis. Although EGFR dysregulation enhances cancer metastasis, the relevant downstream effectors are largely unknown.

MicroRNAs (miRNAs) are small noncoding RNA molecules (about 22 nucleotides in length), which function in RNA silencing and post-transcriptional regulation of gene expression. miRNAs participate in many biological processes, such as cell proliferation, invasion, metastasis, and apoptosis.^13^ Recently, EGFR has been shown to promote prostate cancer bone metastasis by decreasing the expression of miR-1, a tumor suppressor, and increasing the expression of TWIST1, an oncogenic gene.^14^ However, how EGFR promotes cancer metastasis to lungs through microRNAs is unknown.

In this study, we have performed microRNA array analysis and identified miR-338-3p, a tumor suppressor,^15^ as a downstream effector of EGFR. Downregulation of miR-338-3p leads to breast tumor growth and lung metastasis by activating eye absent 2 (EYA2), a member of Eya family that is implicated in cancer cell growth and metastasis.^16, 17^ Clinically, miR-338-3p negatively correlates with the expression of EGFR and EYA2, and EGFR status positively associates with EYA2 expression. miR-338-3p and EYA2 predict lung metastasis when expressed in primary breast cancers.

## Results

### Identification and characterization of miR-338-3p as an EGFR-regulated miRNA in breast cancer

**Figure 1:**
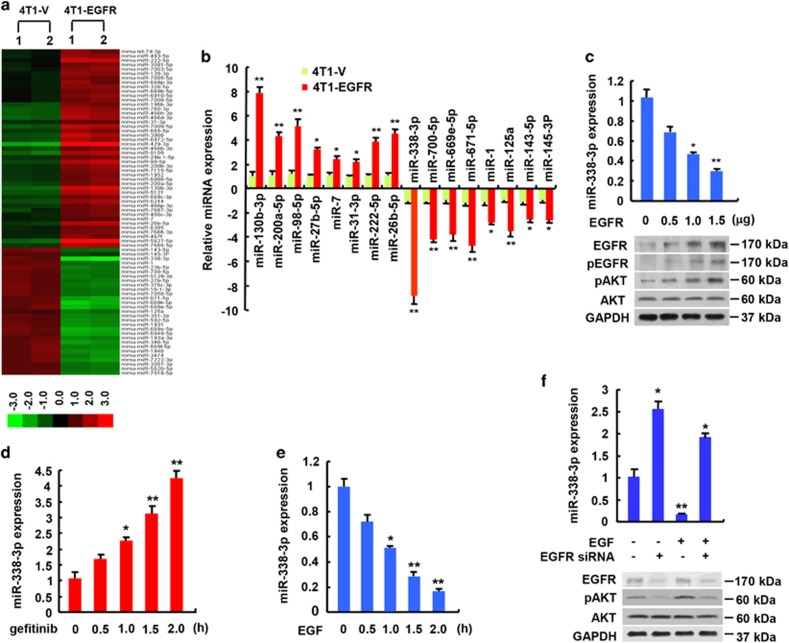
EGFR represses miR-338-3p expression. (**a**) microRNA array analysis shows all differently expressed microRNAs between 4T1 cells stably transfected with EGFR (4T1-EGFR) and empty vector (4T1-V). mmu, mus musculus. (**b**) Verification of some differently expressed microRNAs from A by qRT-PCR. (**c**–**f**) qRT-PCR analysis of miR-338-3p expression in MCF-7 cells transfected with increasing amounts of EGFR (**c**) or in MCF-7 cells treated with the EGFR inhibitor gefitinib (1 *μ*mol/l) (**d**) or EGF (100 ng/ml) (**e**) for the indicated times or in MCF-7 cells transfected with EGFR siRNA and treated with EGF (100 ng/ml) (**f**). Immunoblot with the indicated antibodies is shown (**c**–**f**). GAPDH was used as a loading control. pEGFR, anti-phospho-EGFR (Tyr1068). Values shown are mean±SD of triplicate measurements that have been repeated three times with similar results (**b**–**f**). *P<0.05, **P<0.01 *versus* corresponding control

To investigate EGFR-regulated miRNAs that are potentially involved in human cancer metastasis, we chose 4T1 mouse mammary cancer cell line, whose tumor growth and metastatic spread in BALB/c mice very closely mimic human breast cancer.^18^ This cell line spontaneously produces highly metastatic tumors that can metastasize to the lung, liver, lymph nodes and brain, preferentially the lung, while the primary tumor is growing *in situ*.^19^ We performed miRNA microarray analysis of 4T1 cells stably transfected with EGFR (4T1-EGFR) or empty vector (4T1-V). The total number of mouse miRNAs detected was 1908, of which 74 miRNAs were differentially expressed between 4T1-EGFR and 4T1-V cells (52 miRNAs upregulated and 22 miRNAs downregulated) (Figure 1a). Quantitative RT-PCR confirmed the EGFR-mediated expression of 16 microRNAs, including previously reported miRNAs (e.g. miR-130b, miR-200a and miR-222) identified in Calu-1 lung cancer cells.^20^ (Figure 1b). We found that miR-338-3p was the most downregulated. Thus, we chose miR-338-3p to perform further studies. To further verify the inhibitory effect of EGFR on miR-338-3p, MCF-7 human breast cancer cells and 4T1 cells were transfected with increasing amounts of EGFR (0.5 *μ*g, 1 *μ*g and 1.5 *μ*g). Forty eight hours later, the cells were collected and divided into two parts. One part was used for quantitative RT-PCR analysis and the other part for western blot analysis. As we expected, EGFR expression was gradually increased after transfection with the increasing amounts of EGFR. Importantly, increased EGFR was accompanied by decreased expression of miR-338-3p, suggesting that EGFR represses miR-338a-3p expression in MCF-7 and 4T1 cells in a dose-dependent manner (Figure 1c; Supplementary Figure S1A). In contrast, gefitinib, an EGFR inhibitor, increased miR-338-3p expression (Figure 1d; Supplementary Figure S1B).

EGF is a ligand for EGFR.^21^ Binding of EGF to EGFR induces EGFR-mediated signaling pathways such as PI3K/AKT pathway. Like EGFR overexpression, EGF treatment decreased miR-338-3p expression in MCF-7 and 4T1 cells (Figure 1e; Supplementary Figure S1C). Knockdown of EGFR in MCF-7 and 4T1 cells increased miR-338-3p expression (Figure 1f; Supplementary Figure S1D). Moreover, EGFR knockdown greatly attenuated the ability of EGF to inhibit miR-338-3p expression.

### EGFR inhibits miR-338-3p expression largely through HIF1*α* transcription factor

**Figure 2:**
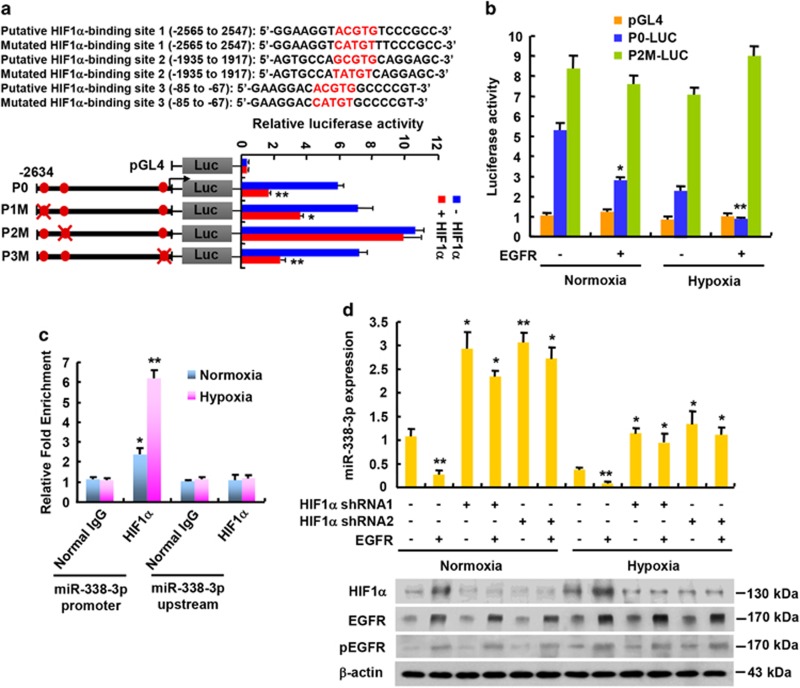
EGFR represses miR-338-3p expression largely through HIF1*α*. (**a**) Luciferase activity of different miR-338-3p promoter reporters in MCF-7 cells transfected with HIF1*α* or empty vector. Filled circles show the position of the putative HIF1*α*-binding site, and the ‘X’ shows the mutated HIF1*α*-binding site. The red letters of each binding region indicate the putative HIF1*α*-binding sequences or the mutated HIF1*α*-binding sequences. (**b**) Luciferase assay of the indicated miR-338-3p promoter reporters from A in MCF-7 cells transfected with EGFR and exposed to either normoxic or hypoxic (1% O_2_) condition. (**c**) ChIP analysis of HIF1*α* occupancy on the miR-338-3p promoter or upstream of the promoter in MCF-7 cells under normoxic or hypoxic condition. (**d**) qRT-PCR analysis of miR-338-3p expression in MCF-7 cells transfected with EGFR or EGFR plus HIF1*α* shRNA1 or HIF1*α* shRNA2 and exposed to either normoxic or hypoxic condition. Representative immunoblot shows the expression of HIF1*α* and EGFR. *β*-actin was used as a loading control. Values shown are mean±S.D. of triplicate measurements that have been repeated 3 times with similar results. *P<0.05, **P<0.01 *versus* corresponding promoter reporter (**a**,**b**). *P<0.05, **P<0.01 *versus* corresponding normal IgG (**c**). *P<0.05, **P<0.01 *versus* corresponding empty vector (**d**)

EGF has been shown to stimulate the expression of hypoxia-inducible factor 1*α* (HIF1*α*), a transcription factor that can promote tumor growth and metastasis.^22, 23^ Hypoxia is one major characteristic of malignant solid tumors and HIF1*α* has a key role in regulation of hypoxic tumor microenvironment. To determine how EGFR represses miR-338-3p expression in breast cancer cells, we performed bioinformatics analysis of miR-338-3p promoter (http://tfbind.hgc.jp). Intriguingly, miR-338-3p promoter contained putative HIF1*α* binding sites. Thus, we tested if EGFR regulates miR-338-3p transcription via HIF1*α*. Indeed, HIF1*α* overexpression decreased the activity of miR-338-3p promoter reporter containing the second putative HIF1*α*-binding site, but not the second mutated HIF1*α*-binding site. The first and third putative HIF1*α*-binding sites had little effect on HIF1*α* repression of miR-338-3p promoter reporter activity (Figure 2a; Supplementary Figure S2A). Moreover, under normoxia, EGFR overexpression inhibited the activity of miR-338-3p promoter reporter containing the second putative HIF1*α*-binding site, but not the second mutated HIF1*α*-binding site, which was more pronounced under hypoxia (Figure 2b; Supplementary Figure S2B). Chromatin immunoprecipitation (ChIP) assay demonstrated that, under normoxia, HIF1*α* was recruited to the region containing the second putative HIF1*α*-binding site within the miR-338-3p promoter, but not to a region upstream of the miR-338-3p promoter, and the recruitment was enhanced under hypoxia (Figure 2c; Supplementary Figure S2C). These data suggest that the second putative HIF1*α*-binding site is the real HIF1*α*-binding site.

Consistent with the results of the miR-338-3p promoter reporter assays, EGFR overexpression activated EGFR phosphorylation and decreased miR-338-3p expression under normoxic and hypoxic conditions (Figure 2d; Supplementary Figure S2D). In contrast, HIF1*α* knockdown increased miR-338-3p expression. Importantly, HIF1*α* knockdown almost abolished the ability of EGFR overexpression to inhibit miR-338-3p expression under normoxia or hypoxia (Figure 2d; Supplementary Figure S2D), suggesting that EGFR represses miR-338-3p expression largely through HIF1*α*.

### MiR-338-3p represses EYA2 expression by targeting its 3′-UTR

**Figure 3:**
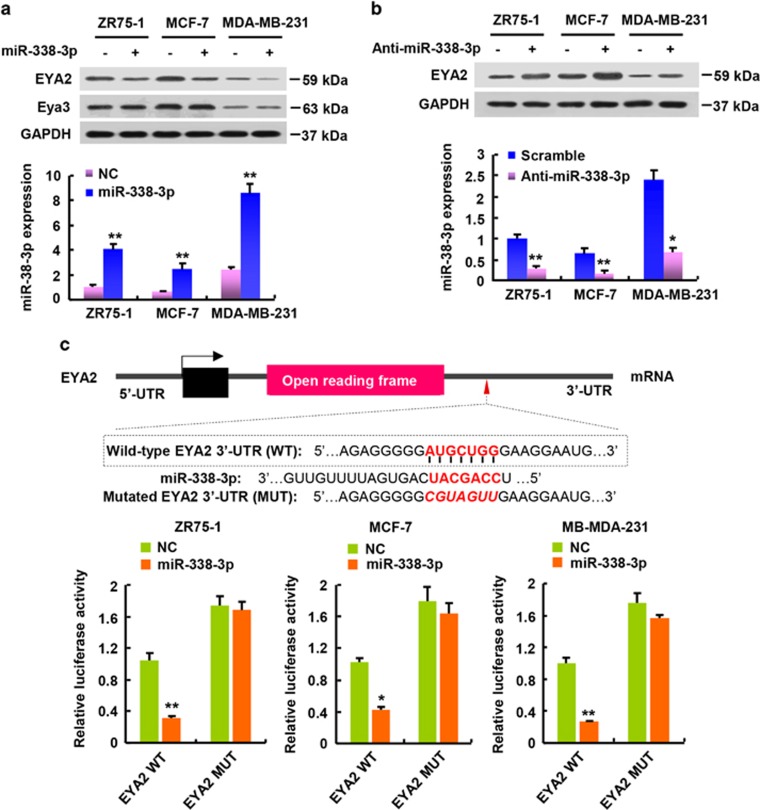
miR-338-3p suppresses EYA2 expression by targeting it’s 3′-UTR. (**a**,**b**) Immunoblot analysis of ZR75-1, MCF-7 and MDA-MB-231 cells transfected with miR-338-3p (**a**) or anti-miR-338-3p (**b**). NC, non-specific control for miRNA-338-3p. Scramble, negative control for anti-miR-338-3p. Histograms under the immunoblots show the corresponding miR-338-3p mRNA expression levels. (**c**) miRNA luciferase reporter assays in the indicated breast cancer cells transfected with miR-338-3p and wild-type or mutated EYA2 reporter. The top panel shows wild-type and mutant forms of putative miR-338-3p target sequences of EYA2 3′-UTR. Red fonts indicate putative miR-338-3p-binding sequences in the EYA2 3′-UTR. Red and italicized fonts indicate mutated miR-338-3p-binding sequences in the EYA2 3′-UTR. WT, wild-type; MUT, mutant. Values shown are mean±S.D. of triplicate measurements that have been repeated 3 times with similar results. *P<0.05, **P<0.01 *versus* corresponding NC or Scramble (**a**,**b**). *P<0.05, **P<0.01 *versus* corresponding EYA2 WT (**c**)

To identify downstream genes regulated by the tumor suppressor miR-338-3p in breast cancer, we used TargetScan and miRanda prediction programs to screen for miR-338-3p-repressed target genes. We found that the EYA2 oncogene might be a potential target as 3′-UTR (untranslated region) of EYA2 contained miR-338-3p-binding site. Indeed, miR-338-3p mimics inhibited EYA2 expression in ZR75-1, MCF-7 and MDA-MB-231 human breast cancer cells and 4T1 mouse cancer cells (Figure 3a; Supplementary Figure S3A). miR-338-3p mimics had little effect on Eya3 expression, indicating that miR-338-3p specifically represses EYA2 expression. On the contrary, the inhibition of a miR-338-3p with an miR-338-3p inhibitor increased EYA2 expression (Figure 3b; Supplementary Figure S3B).

Next, we determined whether the predicted binding site in 3′-UTR of EYA2 was a direct and specific target of miR-338-3p. We performed luciferase reporter assays with wild-type (WT) or mutated EYA2 3′-UTR. miR-338-3p reduced the WT EYA2 3′-UTR reporter activity in ZR75-1, MCF-7, MDA-MB-231 and 4T1 cells (Figure 3c; Supplementary Figure S3C). However, miR-338-3p did not affect the luciferase activity of the mutant reporter in which the binding sites for miR-338-3p were mutated. Taken together, these data suggest that miR-338-3p represses EYA2 expression by directly targeting its 3′-UTR in breast cancer cells.

### EGFR increases EYA2 expression via HIF1*α* repression of miR-338-3p

**Figure 4:**
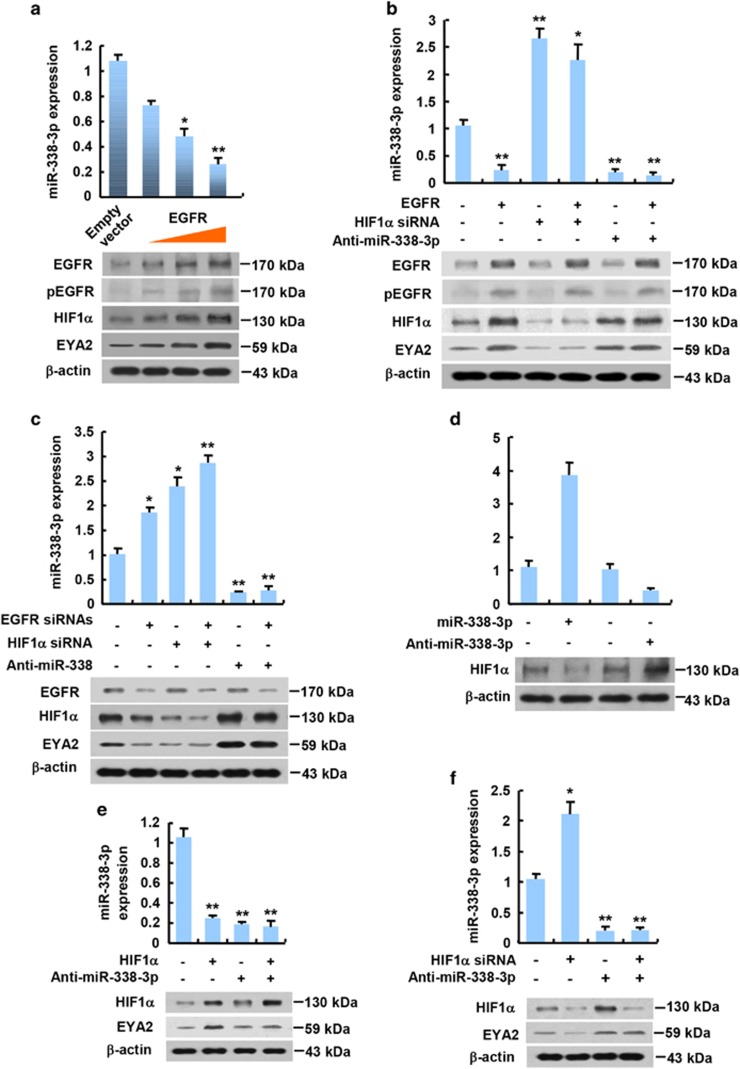
EGFR enhances EYA2 expression via HIF1*α* repression of miR-338-3p. (**a**) qRT-PCR and immunoblot analysis of MCF-7 cells transfected with empty vector or increasing amounts of EGFR. (**b**) qRT-PCR and immunoblot analysis of MCF-7 cells transfected with EGFR or EGFR plus HIF1*α* siRNA or EGFR plus anti-miR-338-3p as indicated. (**c**) qRT-PCR and immunoblot analysis of MCF-7 cells transfected with EGFR siRNAs or EGFR siRNAs plus HIF1*α* siRNA or EGFR siRNAs plus anti-miR-338-3p as indicated. (**d**) qRT-PCR and immunoblot analysis of MCF-7 cells transfected with miR-338-3p mimics or anti-miR-338-3p. (**e**) qRT-PCR and immunoblot analysis of MCF-7 cells transfected with HIF1*α* or HIF1*α* plus anti-miR-338-3p as indicated. (**f**) qRT-PCR and immunoblot analysis of MCF-7 cells transfected with HIF1*α* siRNA or HIF1*α* siRNA plus anti-miR-338-3p as indicated. miR-338-3p expression levels were determined by qRT-PCR (**a**–**e**). The representative immunoblot with the indicated antibodies is shown (**a**–**e**). Values shown are mean±S.D. of triplicate measurements that have been repeated 3 times with similar results. *P<0.05, **P<0.01 *versus* corresponding control

As EGFR inhibits miR-338-3p expression via HIF1*α* and miR-338-3p directly represses EYA2 expression, we tested if EGFR regulates EYA2 expression through HIF1*α*. As expected, EGFR increased HIF1*α* expression and decreased miR-338-3p expression in a dose-dependent manner (Figure 4a; Supplementary Figure S4A). Importantly, EGFR overexpression enhanced EYA2 expression (Figures 4a and b; Supplementary Figures S4A and B), whereas EGFR knockdown reduced EYA2 expression (Figure 4c; Supplementary Figure S4C). However, HIF1*α* knockdown or miR-338-3p inhibition almost abolished the ability of EGFR overexpression or EGFR knockdown to regulate EYA2 expression (Figures 4b and c; Supplementary Figures 4B and C). Consistent with the previously reported results in liver cancer cells,^24^ miR-338-3p mimics inhibited HIF1*α* expression in MCF-7 and 4T1 cells, whereas anti-miR-338-3p increased HIF1*α* expression (Figure 4d; Supplementary Figure S4D). Moreover, HIF1*α* overexpression increased EYA2 expression, while HIF1*α* knockdown decreased EYA2 expression (Figures 4e and f; Supplementary Figures S4E and F). miR-338-3p inhibition abolished the ability of HIF1*α* overexpression or knockdown to regulate EYA2 expression. Taken together, these findings suggest that EGFR promotes EYA2 expression via HIF1*α* inhibition of miR-338-3p.

### EGFR increases breast cancer cell proliferation, migration, invasion and epithelial-to-mesenchymal transition via the miR-338-3p/EYA2 pathway

As EGFR has been shown to promote cancer cell proliferation, migration, and invasion as well as epithelial-to-mesenchymal transition (EMT), a process important for cell migration and invasion,^25, 26^ and EGFR regulates the miR-338-3p/EYA2 axis, we investigated whether EGFR regulates these effects through the miR-338-3p/EYA2 pathway. Cell proliferation assays revealed that EGFR overexpression or miR-338-3p inhibition increased breast cancer cell growth, while EYA2 knockdown decreased breast cancer cell growth (Supplementary Figure S5A). Importantly, miR-338-3p inhibition or EYA2 knockdown greatly attenuated the ability of EGFR to regulate breast cancer cell proliferation. Furthermore, overexpression of miR-338-3p reversed the stimulatory effect of EGFR on breast cancer cell proliferation (Supplementary Figure S5B), and the inhibitory effect of miR-338-3p on breast cancer cell proliferation could be rescued by EYA2 overexpression (Supplementary Figure S5C). Taken together, these data suggest that EGFR increases breast cancer cell proliferation through the miR-338-3p/EYA2 pathway.

**Figure 5:**
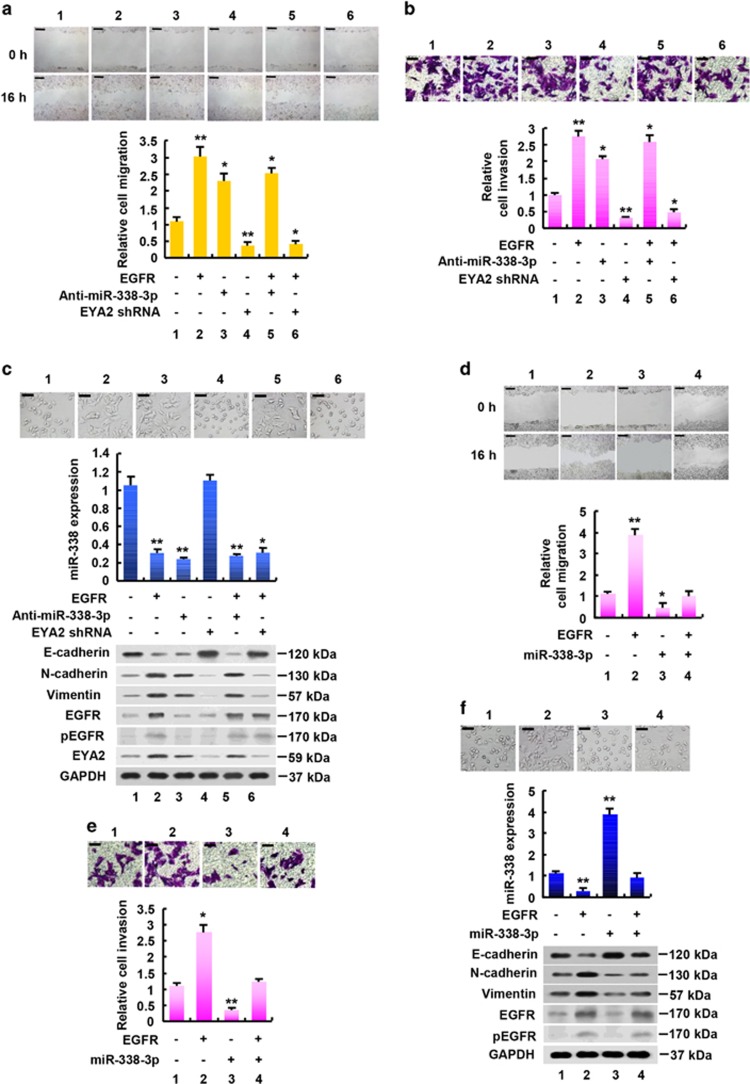
EGFR enhances breast cancer cell proliferation, migration, invasion and EMT via the miR-338-3p/EYA2 pathway. (**a**,**b**) Wound healing (**a**) and invasion (**b**) assays of MCF-7 cells transfected with EGFR or EGFR plus anti-miR-338-3p or EGFR plus EYA2 shRNA as indicated. (**c**) qRT-PCR and immunoblot analyses of MCF-7 cells transfected as in (**a** and **b**). miR-338-3p expression levels were determined by qRT-PCR. The representative immunoblot with the indicated antibodies is shown. Morphologic changes are shown in the photographs. (**d**,**e**) Wound healing (**d**) and invasion (**e**) assays of MCF-7 cells transfected with EGFR or EGFR plus miR-338-3p as indicated. (**f**) MCF-7 cells were transfected as in (**d** and **e**), and analyzed by qRT-PCR and immunoblot as in c. All values shown are mean±S.D. of triplicate measurements and have been repeated 3 times with similar results. *P<0.05, **P<0.01 *versus* corresponding control. Scale bar: 100 *μ*m (**a**–**f**)

Next, we determined whether EGFR promotes breast cancer cell migration, invasion and EMT via the miR-338-3p/EYA2 axis. As expected, EGFR enhanced breast cancer cell migration and invasion (Figures 5a and b). miR-338-3p inhibition or EYA2 knockdown greatly attenuated the ability of EGFR to enhance breast cancer cell migration and invasion. EGFR overexpression or miR-338-3p inhibition promoted morphologic changes from a polarized epithelial phenotype to an elongated fibroblastoid phenotype, while EYA2 knockdown had opposite effects (Figure 5c), suggesting that EGFR overexpression and miR-338-3p inhibition promotes EMT and EYA2 knockdown suppresses EMT. Indeed, EGFR overexpression or miR-338-3p inhibition decreased expression of the epithelial marker E-cadherin and increased that of the mesenchymal markers N-cadherin and Vimentin, whereas EYA2 knockdown increased expression of E-cadherin and decreased that of N-cadherin and Vimentin (Figure 5c). Importantly, miR-338-3p inhibition or EYA2 knockdown greatly attenuated the ability of EGFR to regulate the morphologic changes and the expression of the EMT markers. Furthermore, miR-338-3p overexpression reversed the stimulatory effect of EGFR on breast cancer cell migration and invasion as well as EMT (Figures 5d–f), and the inhibitory effect of miR-338-3p on these phenotypes could be rescued by EYA2 overexpression (Supplementary Figures S6A–C). Taken together, these results suggest that EGFR increases breast cancer cell migration and invasion as well as EMT through the miR-338-3p/EYA2 pathway.

### EGFR promotes tumor growth and lung metastasis through the miR-338-3p/EYA2 pathway

**Figure 6:**
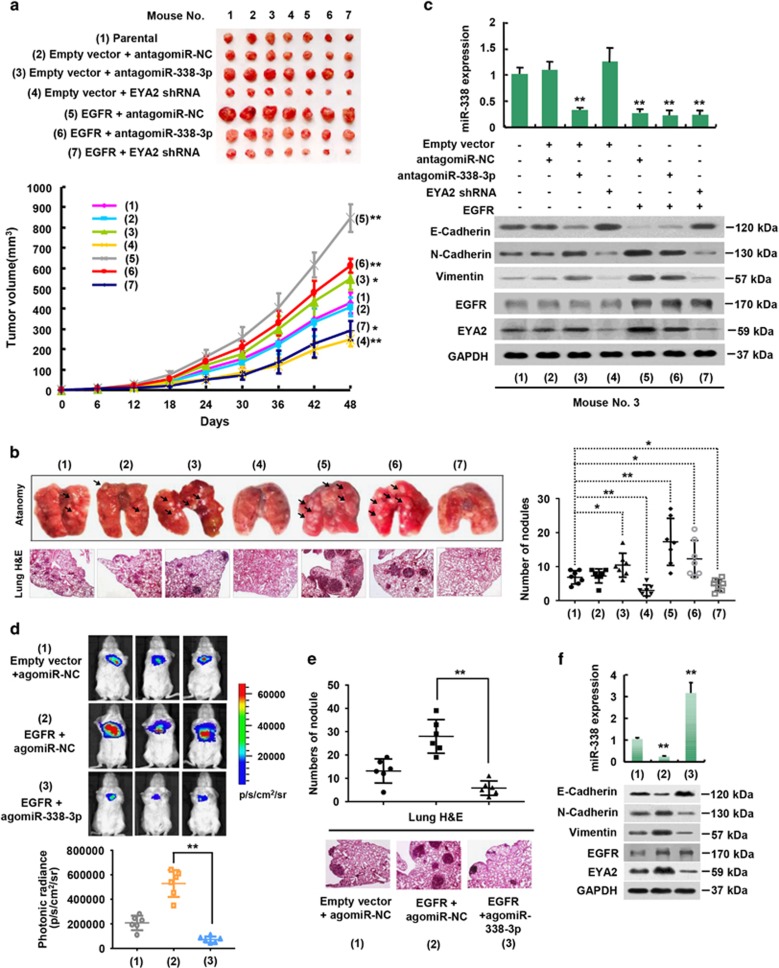
EGFR promotes breast tumor growth and metastasis through the miR-338-3p/EYA2 pathway. (**a**) 4T1 xenograft tumors of the indicated groups were established in BABL/c mice. At the indicated times, tumors were measured (mean±S.D.; *n*=7). (**b**) Representative H&E-stained sections of the lung tissues from (**a**) are shown. The number of tumor nodules was examined under an anatomical microscope. Symbols represent individual mice; horizontal bars indicate the mean±S.D. (**c**) qRT-PCR and immunoblot analyses of representative excised tumors from (**a**). miR-338-3p expression levels were determined by qRT-PCR. The representative immunoblot with the indicated antibodies is shown. (**d**) Breast cancer cell metastases were established in nude mice (*n*=6) by tail vein injection of 4T1 cells stably expressing firefly luciferase and EGFR or empty vector. On the second day, agomiR-338-3p or scramble was injected via tail vein. Bioluminescence images were collected at 30 days. The luminescence signal is represented by an overlaid false-color image with the signal intensity indicated by the scale. (**d**) Representative H&E-stained sections of the lung tissues from d are shown. The number of tumor nodules was analyzed as in (**c**). (**f**) The representative excised lung tissues from d were analyzed by qRT-PCR and immunoblot as in b. *P<0.05, **P<0.01 *versus* corresponding control

To confirm the *in vitro* phenotype of the EGFR/miR-338-3p/EYA2 pathway, we first investigated the effect of the pathway on breast tumor growth by injecting 4T1 cells harboring the indicated constructs and/or the miR-338-3p inhibitor into the mammary fat pads of BALB/c mice. As expected, EGFR overexpression markedly increased breast tumor growth (Figure 6a). Similar trend was obtained when miR-338-3p was inhibited. On the contrary, tumor growth was suppressed when EYA2 was knocked down. However, the effect of EGFR overexpression on tumor growth was dramatically attenuated when miR-338-3p was inhibited or EYA2 was knocked down (Figure 6a).

Next, we examined the effect of the pathway on breast cancer metastasis. The number of the nodules spread throughout the pulmonary region was markedly increased in the EGFR-expressing group compared with that in empty vector group (Figure 6b). miR-338-3p inhibition had similar effect. In contrast, EYA2 knockdown led to decreased metastatic spread of breast cancer cells to the lung. Importantly, miR-338-3p inhibition or EYA2 knockdown greatly attenuated the ability of EGFR to regulate lung metastasis (Figure 6b). Histologic analysis on the lungs confirmed the metastasis foci. Consistent with the results of the tumor metastasis experiments, miR-338-3p inhibition or EYA2 knockdown markedly attenuated the ability of EGFR to regulate the EMT markers (Figure 6c).

To investigate therapeutic effect of miR-338-3p on breast cancer lung metastasis experimentally, we injected 4T1 cells stably expressing firefly luciferase and EGFR or empty vector via mouse tail vein. Bioluminescence imaging and H&E staining showed that treatment with agomiR-338-3p, a modified miR-338-3p, which was more stable than miR-338-3p mimics, greatly reduced EGFR-mediated breast cancer lung metastasis (Figures 6d and e). This was accompanied by increased E-cadherin and decreased N-cadherin, Vimentin and EYA2 (Figure 6f).

### Correlation among EGFR, miR-338-3p and EYA2 and correlation of miR-338-3p and EYA2 with lung metastasis in human breast cancer patients

**Figure 7:**
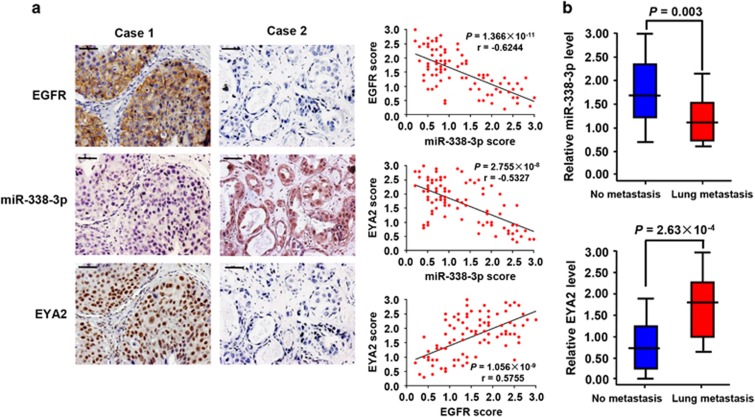
Correlation among EGFR, miR-338-3p and EYA2 and correlation of miR-338-3p and EYA2 with lung metastasis in human breast cancer patients. (**a**) The relationship among EGFR, miR-338-3p and EYA2 expression was detected by Spearman rank correlation analysis in breast cancer samples. Symbols represent individual samples. EGFR and EYA2 expression was examined by immunohistochemistry and miR-338-3p by miRNA *in situ* hybridization. Case 1 represents high EGFR, low miR-338-3p and high EYA2 expression, and case 2 low EGFR, high miR-338-3p and low EYA2 expression. Scale bar: 100 *μ*m. (**b**) Comparison of miR-338-3p or EYA2 scores in breast cancer patients with and without lung metastasis (independent *t*-test)

We assessed EGFR and EYA2 expression by immunohistochemical staining (IHC) and miR-338-3p expression by miRNA *in situ* hybridization (MISH) in 95 human breast cancer samples. In agreement with EGFR inhibition of miR-338-3p and miR-338-3p inhibition of EYA2 in cultured cells, the expression of miR-338-3p negatively correlated with EGFR or EYA2 expression (Figure 7a). Consistent with EGFR promotion of EYA2 in cultured cells, the expression of EGFR positively associated with EYA2 expression. Moreover, low miR-338-3p expression and high EYA2 expression in primary breast cancer correlated with lung metastasis (Figure 7b). We confirmed the specificity of the EGFR and EYA2 antibodies by IHC of breast cancer tissues or immunoblot with cell lysates (Supplementary Figures S7A and B) and that of miR-338-3p staining by correlation analysis of different miR-338-3p expression in breast tissues examined by hybridization and quantitative RT-PCR, respectively (Supplementary Figures S7C and D).

## Discussion

EGFR overexpression occurs in many solid tumors, and is correlated with tumor progression and metastasis, poor prognosis, and resistance to chemo- and radiotherapy. Several studies have shown an inverse correlation between EGFR expression and disease-free and overall survival of breast cancer.^7, 8, 9, 10^ It is well established that EGFR activates two critical signaling pathways, the MAPK/ERK1/2 and PI3K/AKT pathways, leading to increased cancer cell proliferation and metastasis.^1, 2, 3^ In this study, we identify a novel HIF1*α*/miR-338-3p/EYA2 axis that controls EGFR-mediated tumor growth and metastasis (Supplementary Figure S8). EGFR promotes breast tumor growth and lung metastasis by HIF1*α* repression of miR-338-3p and subsequent activation of EYA2. EGF has been shown to stimulate the expression of HIF1*α*.^23, 24^ Consistent with this finding, we demonstrated that overexpression of EGFR, the EGF receptor, increases HIF1*α* expression. We further showed that miR-338-3p is a new transcriptional target of HIF1*α* and EYA2 is a novel direct target of miR-338-3p.

miR-338-3p has been shown to act as a tumor suppressor.^27, 28, 29, 30^ miR-338-3p is downregulated in various cancers, including breast cancer, gastric cancer, ovarian cancer, colorectal carcinoma and lung cancer. Low expression of miR-338-3p is associated with poor clinical outcome. Overexpression of miR-338-3p was demonstrated to inhibit cancer cell proliferation, migration and invasion. miR-338-3p represses EMT in gastric cancer cells by targeting ZEB2 and MACC1/Met/Akt signaling.^28^ miR-338-3p decreases gastric cancer cell proliferation, migration and invasion as well as EMT by inhibiting NRP1 expression.^31^ In hepatoma carcinoma cells, miR-338-3p suppresses EMT and metastasis via both inhibition of the SHH/Gli1 pathway and direct binding of N-cadherin.^32^ miR-338-3p represses lung cancer cell EMT and metastasis by inhibiting Sox4 transcription factor.^33^ EYA2 expression has been shown to be overexpressed in a variety of cancers, including breast cancer, ovarian cancer and lung cancer, and its overexpression is correlated with poor prognosis.^34, 35, 36^ EYA2 increases proliferation, migration, invasion, and metastasis in breast cancer cells. EYA2 is also involved in stimulation of EMT, a process important for cancer cell migration, invasion, and metastasis.^37^ The EYA2 gene is hypomethylated in lung cancer cells, resulting in EYA2 overexpression.^36^ We showed that miR-338-3p directly inhibited EYA2 expression at the post-transcriptional level in cultured cancer cells and its expression negatively correlated with EYA2 expression in breast cancer patients. Moreover, we demonstrated that, through EYA2 inhibition, miR-338-3p not only suppressed breast cancer cell proliferation, EMT, migration and invasion *in vitro*, but also prevented breast cancer cell metastasis to lung *in vivo*. Thus, miR-338-3p activation may be useful for treatment of cancer with EYA2 overexpression.

The majority of cancer deaths are due to the development of metastatic disease.^12^ Lung and bone are frequent sites of breast cancer metastasis.^38^ Chang *et al.* reported that EGFR enhances prostate cancer bone metastasis by decreasing miR-1 expression and increasing TWIST1 expression *in vitro* and in nude mice, and decreased miR-1 levels associate with enhanced EGFR and TWIST1 expression in a cohort of human prostate cancer specimens.^14^ In our murine metastatic model, EGFR overexpression promoted breast cancer lung metastasis, and miR-338-3p inhibition or EYA2 knockdown almost abolished the ability of EGFR to enhance lung metastasis of breast cancer, suggesting that EGFR increases breast cancer lung metastasis by regulating the expression of miR-338-3p and EYA2. In addition, we showed that EGFR inhibits the expression of miR-338-3p and decreases that of EYA2 in cultured breast cancer cells. Clinically, miR-338-3p expression negatively correlates with the expression of EGFR and EYA2, and low miR-338-3p levels and high EYA2 levels associate with breast cancer lung metastasis. Our current study provides a molecular explanation linking high EGFR levels in breast cancer with downregulated miR-338-3p and upregulated EYA2, and suggests that the EGFR/miR-338-3p/EYA2 pathway determines breast cancer lung metastasis and that miR-338-3p and EYA2 may be used to identify primary breast tumors with the capacity of metastasis, especially lung metastasis.

EGFR inhibition has been used as a strategy for treatment of non-small-cell lung cancer, pancreatic cancer, breast cancer, colon cancer and some other cancers. There are several kinds of drugs against EGFR, including EGFR tyrosine kinase inhibitors (e.g. gefitinib) and EGFR monoclonal antibody (e.g., cetuximab).^39, 40, 41^ Although targeted therapy has benefitted many breast cancer patients, the combination therapy is still recommended for treatment of recurrent and metastatic patients. On the other hand, resistance to drugs against EGFR has been a major clinical problem.^42^ We demonstrated that mouse tail vein injection of agomiR-338-3p, a modified miR-338-3p mimics, significantly decreased the incidence of breast cancer metastasis to the lung. Recently, the inhibitors of EYA2, a member of a class of protein tyrosine phosphatases, have been experimentally investigated.^43, 44^ Therefore, miR-338-3p activation or EYA2 inhibition or combination therapy targeting EGFR/miR-338-3p/EYA2 axis may be a promising way to treat patients with metastatic cancer.

## Materials and Methods

### Cell lines, plasmids, RNA oligonucleotides, Lentivirus and reagents

Human breast cancer cell lines MCF-7, ZR75-1 and MDA-MB-231 were purchased from the American Type Culture Collection (Manassas, VA, USA). 4T1 cells labeled with firefly luciferase was a kind gift from Professor Xiaodan Yu at Beijing Institute of Basic Medical Sciences. The EGFR expression vector has been described previously.^45^ The eukaryotic expression vectors were generated by inserting PCR-amplified fragments into pcDNA3 (Invitrogen, Carlsbad, CA, USA). Prokaryotic plasmids encoding GST-proteins were constructed in pGEX-KG (Amersham Pharmacia Biotech, Stockholm, Sweden). The miR-338-3p promoter and its mutant luciferase reporters were made by inserting PCR-amplified promoter fragments from genomic DNA into the pGL4-Basic vector (Promega, Madison, WI, USA). The cDNA target sequences of siRNAs and/or shRNAs for EGFR, HIF1*α* and EYA2 are listed in Supplementary Table 1. Anti-EYA2 and anti-HIF1*α* were purchased from Sigma-Aldrich (St. Louis, MO, USA). Anti-EGFR, anti-Vimentin, anti-GAPDH and anti-*β* actin were purchased from Santa Cruz Biotechnology (Dallas, TX, USA). Anti-phospho-EGFR (Tyr1068) was purchased from Cell Signaling Technology (Danvers, MA, USA). Anti-E-cadherin, anti-N-cadherin were obtained from BD Biosciences (Franklin Lakes, NJ, USA). miR-338-3p mimics was purchased from GenePharma (Suzhou, China). The cholesterol-modified agomiR-338-3p and antagomiR-338-3p (anti-miR-338-3p) and their respective negative control miRNAs were purchased from RiboBio. Lentivirus was produced by cotransfection of HEK293T cells with recombinant lentivirus vectors and pPACK Packaging Plasmid Mix (System Biosciences, Palo Alto, CA, USA) using Megatran reagent (Origene, Rockville, MD, USA), and were used to infect target cells according to according to the manufacturers’ instructions. Lipofectamine 2000 reagent and Lipofectamine RNAiMAX were purchased from Invitrogen.

### Cell culture and cell treatment

MCF-7, ZR75-1, MDA-MB-231 and 4T1 cells were cultured in Dulbecco’s modified Eagle’s medium (DMEM) (GIBCO, Carlsbad, CA, USA) supplemented with 10% fetal bovine serum at 37  °C and 5% CO_2_. Cells were grown to about 80% confluency for use. For EGF treatment experiments, MCF-7 and 4T1 cells were serum starved overnight, and then treated with 100 ng/ml EGF (Sigma-Aldrich) for 0.5 h, 1 h, 1.5 h and 2 h at 37  °C.

### microRNA microarray analysis

Total RNA was extracted from 4T1 cells expressing EGFR or empty vector by using TRIzol (Invitrogen) according to the manufacturer's protocol. RNA was then labeled with biotin using Genisphere FlashTag labeling kit (Genisphere, Hatfield, PA, USA) and hybridizated on microRNA microarray chips (CapitalBio). Briefely, 1 *μ*g of RNA was labeled with biotin during reverse transcription. Hybridization was performed on Affymetrix GeneChip miRNA 4.0 chip which contains 30 424 probes for mature microRNAs of all species, among which 1908 probes for mouse mature microRNAs. Hybridization signals were detected by GeneChip Scanner 3000. Images were analyzed by Affymetrix GeneChip Command Console (AGCC) Software. All data were analyzed by Affymetrix Expression Console Software 1.3. For each miRNA, fold changes were calculated, and fold change ≥2 represents genes upregulated and fold change ≤0.5 represents genes downregulated.

### Quantitative reverse transcription-PCR

Total RNA was isolated using TRIzol reagent according to the manufacturer's protocol (Invitrogen). RNA was reverse transcribed into cDNA by miRcute miRNA First-Strand cDNA Synthesis Kit (Tiangen, Beijing, China). Real-time PCR was performed with primers listed in Supplementary Table 2.

### Transfection and luciferase reporter assay

Cells were cultured in 24-well plates. Lipofectamine 2000 reagent and Lipofectamine RNAiMAX were used for transfections of plasmids and/or siRNAs/shRNAs according to the manufacturer’s instructions (Invitrogen). Luciferase activity was determined using a luciferase assay kit (Promega) 48 h after transfection according to the manufacturer’s protocol.

### ChIP assay

ChIP assay was performed using the Magna ChIP Assay Kit (Millipore) according to the manufacturer’s protocol. Quantitative analysis was carried out by real-time PCR with primers listed as follows: human miR-338-3p promoter sense, 5′-CCGTGGGTGATGCTGTCTG-3′ human miR-338-3p promoter antisense, 5′-CAGCACAGGCCTTTGTGCC-3′ human miR-338-3p upstream sense, 5′-CAGCCACCCACTCAGAGCG-3′ human miR-338-3p upstream antisense, 5′-GTGGCTCTGAGAATCTTCG-3′ mouse miR-338-3p promoter sense, 5′-CAGCGTGCAGGAGCAGATGC-3′ mouse miR-338-3p promoter antisense, 5′-GCCCTGGAAGAGTCCACGGG-3′ mouse miR-338-3p upstream sense, 5′-CAGCGTGGGAGCAGAATTCA-3′ mouse miR-338-3p upstream antisense, 5′-GCCTTTGTTG GGGGCTGCTT-3′.

### Cell proliferation, migration and invasion assays

Cell proliferation was detected by the CCK-8 Kit (Dojindo Laboratories) according to the manufacturer’s protocol. Wound healing assay was applied to measure cell migration. Briefly, cells were grown as confluent monolayers in six-well plates. The cell monolayers were scratched with a 200 *μ*l pipette tip and washed twice with PBS. Cells were then cultured for 16 h to generate would closure. Cell invasion assays were performed with Matrigel Invasion Chambers (BD Biosciences) following the manufacturer's introduction. Briefly, cells were placed on the upper surface of the transwell insert. After 16 h, the invasive cells were fixed with 4% paraformaldehyde and stained with 0.5% crystal violet. The number of invasive cells were counted in five randomly selected microscope visions and photographed.

### Analysis of tumor growth and metastasis *in vivo*

Animal studies were approved by the Institutional Animal Care Committee of Beijing Institute of Biotechnology. For tumor growth and metastasis study,^46^ BALB/c female mice (4–6-week-old) were randomly divided into seven groups (*n*=7). The seven experimental groups are as follows. 3 × 10^6^ 4T1 cells stably expressing EGFR or empty vector were treated with antagomiR-338-3p (250 nmol) or antagomiR-NC (250 nmol), a negative control for three days. 3 × 10^6^ 4T1 cells stably expressing EGFR or empty vector were infected with pLenti-H1 EYA2 shRNA lentivirus. The parallel experimental group was set. The cells for the seven groups were inoculated into the second mammary fat pad on the right side of BALB/c mice. Four weeks after tumor cell inoculated, antagomiR-338-3p (10 nmol) or antagomiR-NC (10 nmol) were intratumorally injected into appropriate groups (the groups with the antagomiRs) twice a week for 2 weeks. Tumor size was measured at the indicated times using calipers. Tumor volume was estimated according to the following formula: volume=(longest diameter × shortest diameter^2^)/2. The mice were sacrificed at the indicated time. Excised tumors were frozen in liquid nitrogen for further study. Cancer metastasis was examined by H&E staining.

For lung metastasis study, 1 × 10^7^ 4T1 cells labeled with firefly luciferase and expressing EGFR or empty vector were treated for three days with agomiR-338-3p (1 *μ*mol) or agomiR-NC (1 *μ*mol), a negative control. The treated cells (1 × 10^6^) were injected into the lateral tail vein of each BALB/c female mouse. Four weeks later, the animals were imaged using the IVIS200 imaging system (Xenogen Corporation, Alameda, CA, USA). After sacrification, all the lungs were excised for metastatic foci analysis.

### Clinical samples, miRNA *in situ* hybridization and immunohistochemistry

Ninety five breast cancer samples used were obtained from Chinese PLA General Hospital with the informed consent of patients and with the approval of the Institutional Review Committees of Chinese PLA General Hospital. All patients were female with 28–79 years of age (mean age: 52.9 years).

miRNA *in situ* hybridization (MISH) was performed on paraffin tissue sections using 5′ and 3′ double digoxigenin (DIG)-labeled microRNA detection probes according to the manufacturer’s instructions (Exonbio). The sequence of miR-338-3p probe is 5′-CAACAAAATCACTGATGCTGGA-3′, complementary to miR-338-3p. The sequence of U6 probe as a positive control is 5′-GAACGCTTCACGAATTTGCGTGTCATCCTTGCGCA-3′. The sequence of scramble probe as a negative control is 5′-GTGTfAACACGTCTATACGCCCA-3′. Briefly, the paraffin tissue sections were hybridized with DIG-labeled probe at 42 °C for 48 h and then were stained with DAB. The sections were dehydrated in increased concentration of ethanol series, cleared in xylene and mounted with neutral resin.

Formalin fixed and paraffin embedded tissue sections were subjected to immunohistochemistry (IHC) as previously described.^47^ Anti-EGFR antibody (ZSGB-BIO) in working solution for IHC was used directly. Anti-EYA2 antibody (Sigma-Aldrich) was used at a dilution of 1:100 for IHC. The miR-338-3p, EGFR or EYA2 score was generated by multiplying the percentage of stained cells (0–100%) by the intensity of the staining (negative, 0; low, 1+ medium, 2+ strong, 3+). Thus, the score is between 0 and 3. In detail, a semi-quantitative scoring system was used as previous described.^48^ Each slice was observed with five random fields under a light microscope (× 20 magnification). The stained cells/total cells were counted. The percentage of positive cells (0–100%) was calculated. The average of the five calculated results was taken. The level of expression was also assessed by light microscope. Staining intensity was determined by the depth of the color and divided into four grades: no staining (0), light brown (1), brown (2) and dark brown (3). Each slice was also observed with five random fields under a light microscope (× 20 magnification). The mean of the five fields staining intensity was taken. Staining was scored independently by two pathologists who were blinded to each other’s findings. All conflicting calls on scoring were adjudicated by a third individual.

### Statistics

All the experiments *in vitro* were performed in triplicate and repeated 3 times. Statistical significance in cell proliferation, migration and invasion assays as well as luciferase reporter assays was determined by two-tailed Student’s *t*-test. The statistical analyses were calculated by the SPSS 17.0 statistical software package. *P* values of less than 0.05 were considered statistically significant. The correlation among the expression of miR-338-3p, EGFR and EYA2 was examined by Spearman rank analysis using GraphPad PRISM 7.
