# Supplementary material for: The EGFR/miR-338-3p/EYA2 axis controls breast tumor growth and lung metastasis
Source: Cell Death Dis. 2017 Jul 13;8(7):e2928–. doi: 10.1038/cddis.2017.325 (PMC5550870; doi:10.1038/cddis.2017.325)
Supplement: Supplementary Figures [file cddis2017325x1.pdf]

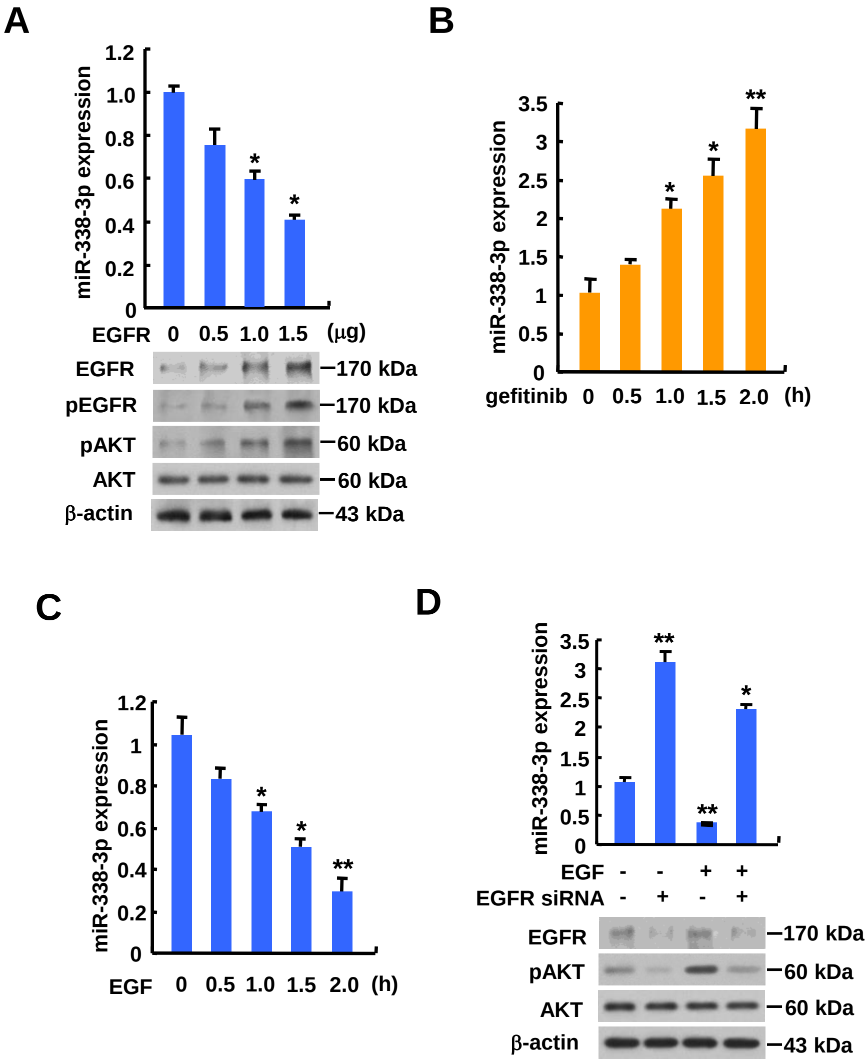


Liang YC et al, Supplementary Figure S1

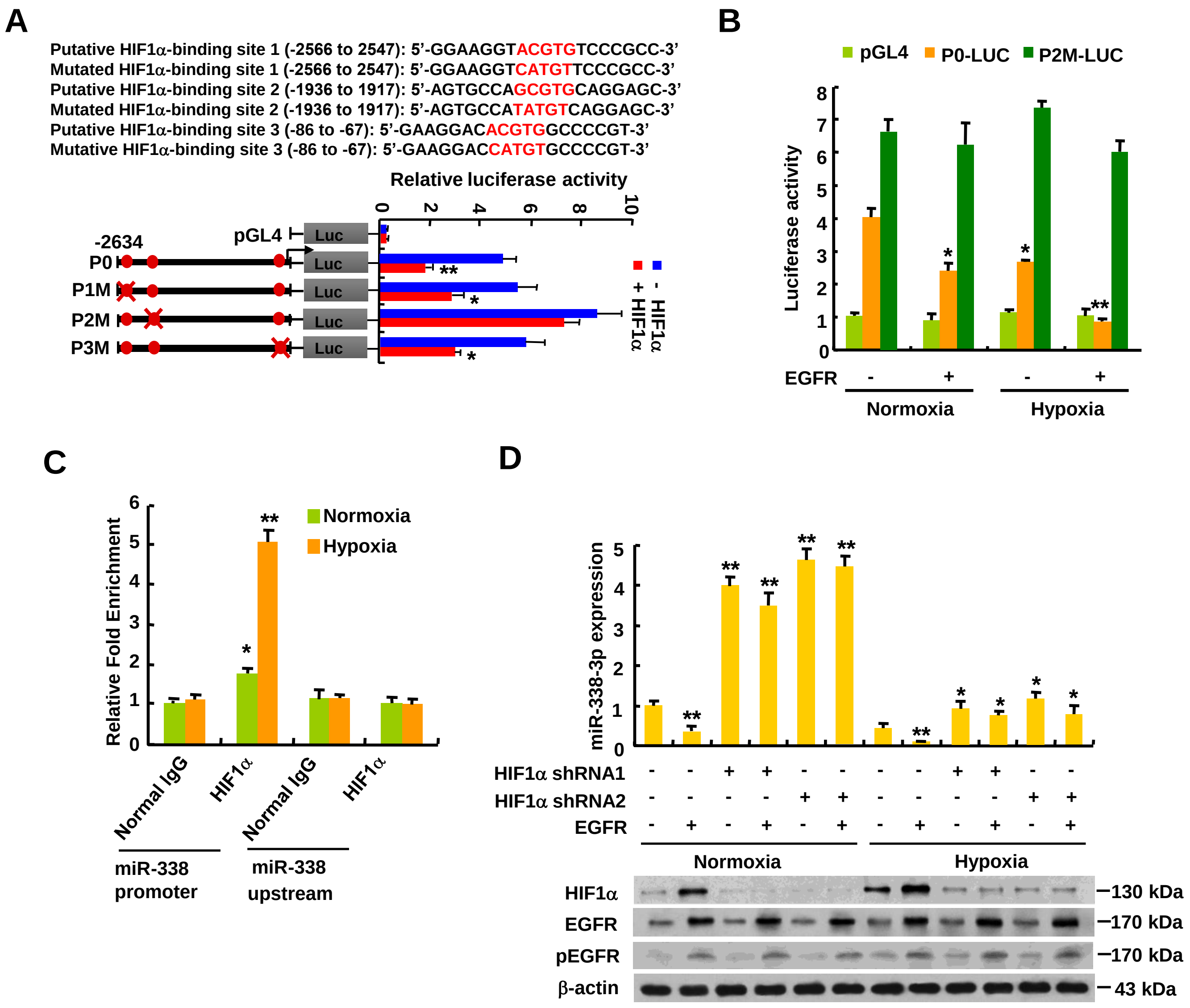


Liang YC et al, Supplementary Figure S2

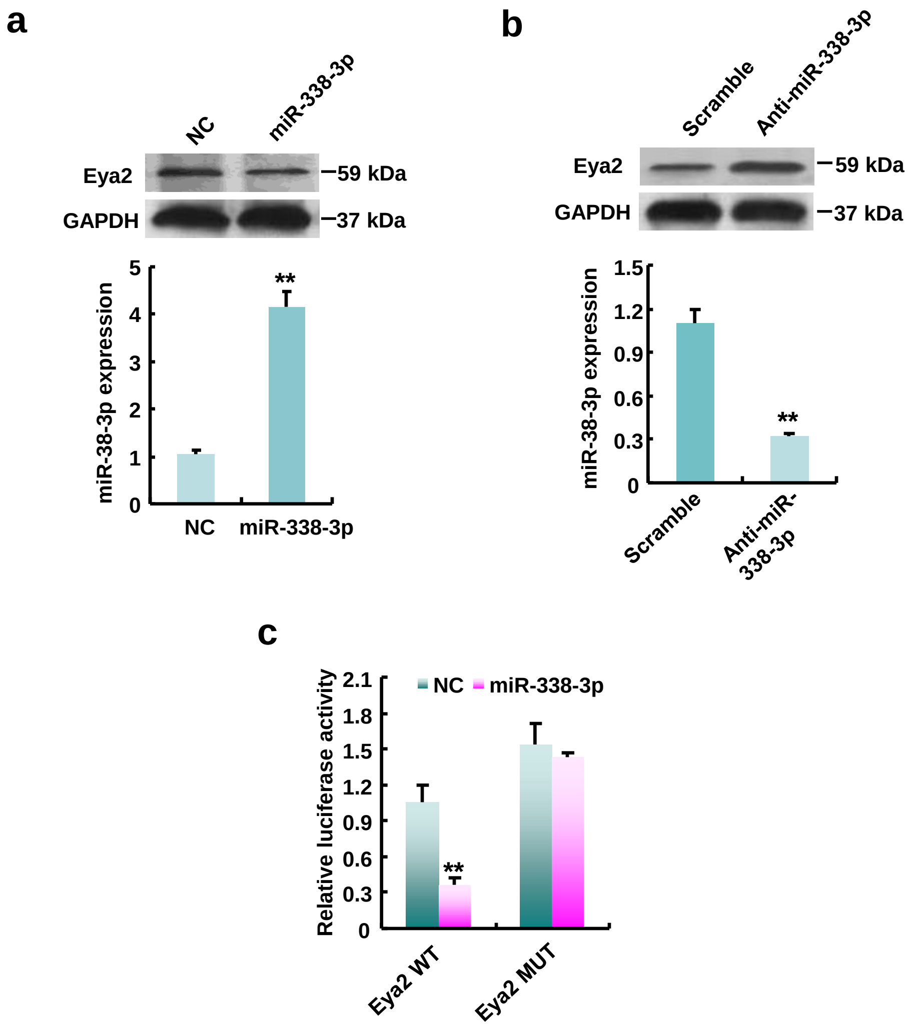


Liang YC et al, Supplementary Figure S3

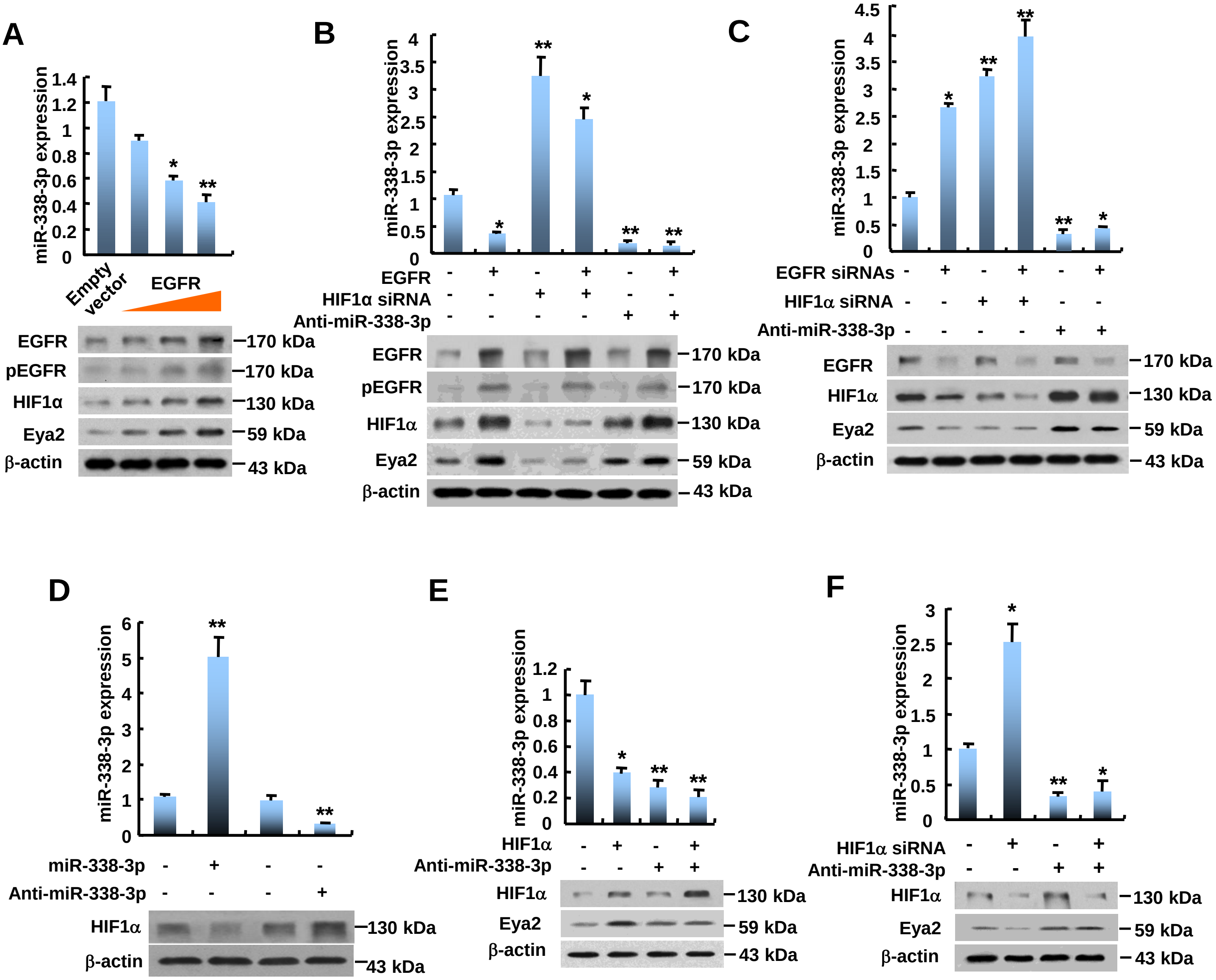


Liang YC et al, Supplementary Figure S4

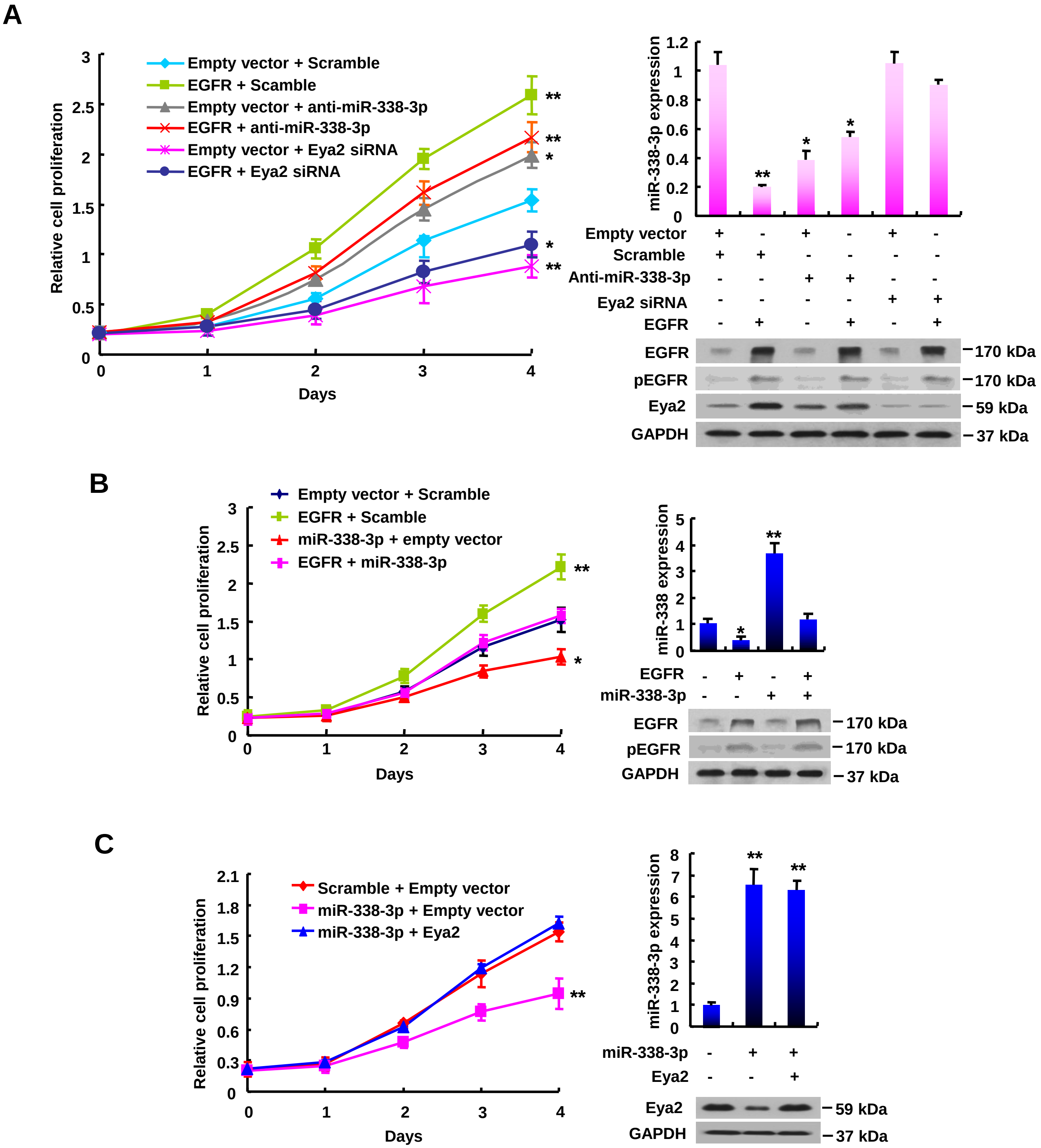


Liang YC et al, Supplementary Figure S5

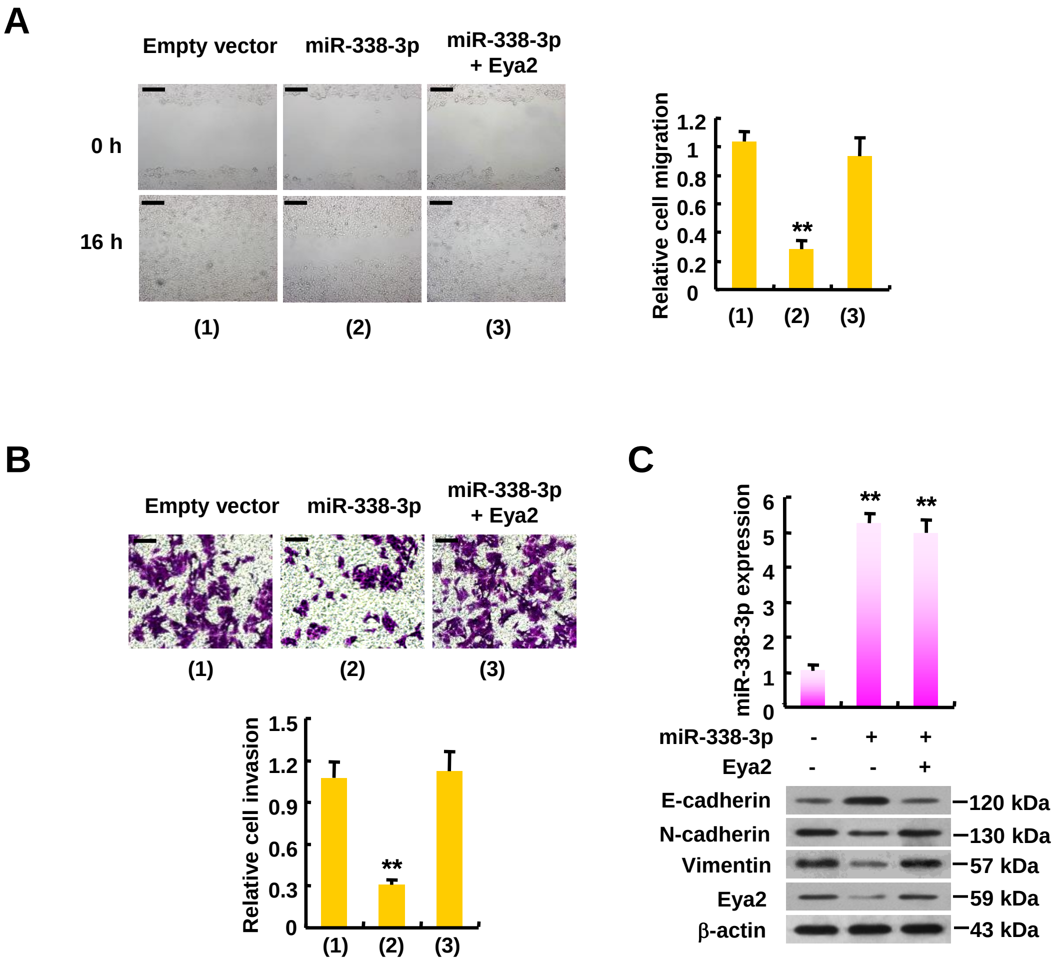


Liang YC et al, Supplementary Figure S6

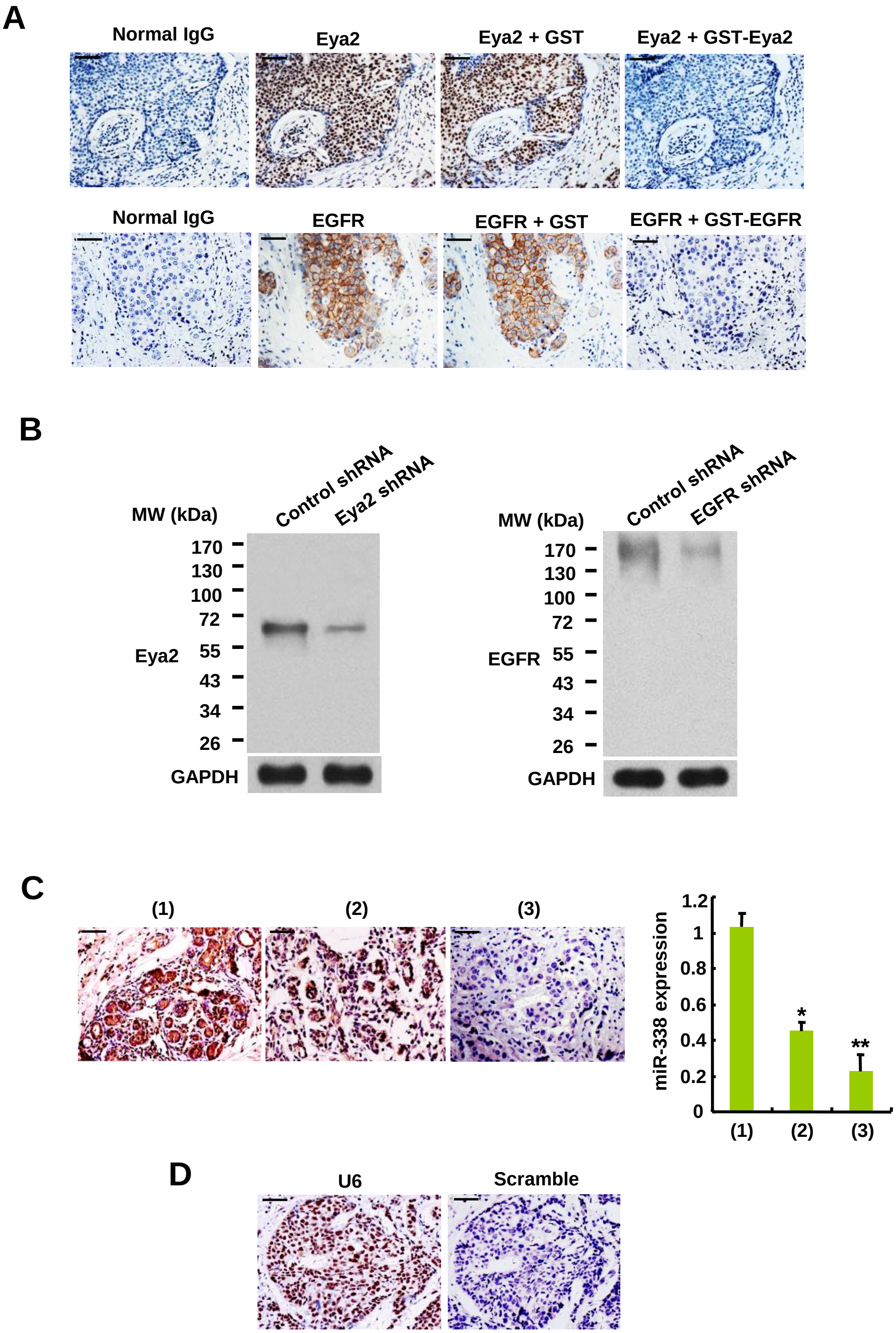


Liang YC et al, Supplementary Figure S7

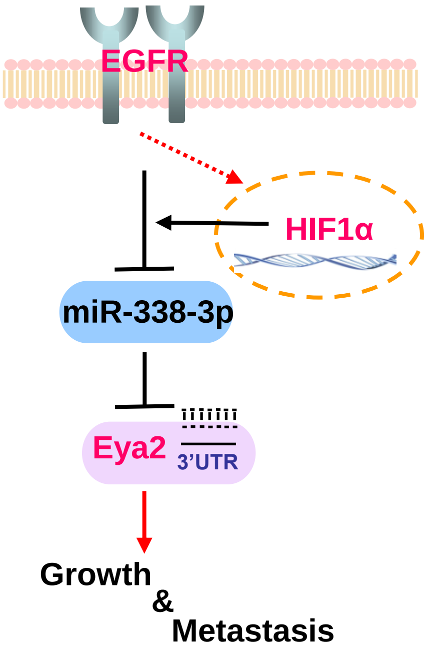


Liang YC et al, Supplementary Figure S8
