# Supplementary material for: The EGFR/miR-338-3p/EYA2 axis controls breast tumor growth and lung metastasis
Source: Cell Death Dis. 2017 Jul 13;8(7):e2928–. doi: 10.1038/cddis.2017.325 (PMC5550870; doi:10.1038/cddis.2017.325)
Supplement: Supplementary Table 1 [file cddis2017325x2.doc]

**Supplementary Table S1. The cDNA target sequences of siRNAs or shRNAs**

| **Gene** | **Target sequence (5’→3’)** |
| --- | --- |
| Hsa-HIF1α (siRNA) [1]  Hsa-EGFR (siRNA) [2]  Hsa-EGFR (siRNA) [2]  Hsa-Eya2 (siRNA) [3]  Hsa-HIF1α (shRNA) [4]  Hsa-HIF1α (shRNA) [5]  Hsa-Eya2 (shRNA) [6]  Mus-HIF1α (siRNA) [1]  Mus-EGFR (siRNA) [7]  Mus-EGFR (siRNA) [7]  Mus-Eya2 (siRNA)  Mus-HIF1α (shRNA) [8]  Mus-HIF1α (shRNA) [8]  Mus-Eya2 (shRNA) | CCTACTGCAGGGTGAAGAA  CGCAAAGTGTGTAACGGAATA  CTGACTCCGTCCAGTATTGAT  CAGCGAUU GUCUGGAUAAA  GGGTAAAGAACAAAACACA  GTGATGAAAGAATTACCGAAT  CATACCAACCTACTGCAGA  CCTACTGCAGGGTGAAGAA  AATGGACTTACAGAGCCATCC  AAAGAAGACGCCTTCTTGCAG  GAGCCCCTACACCTACCC  CTAGAGATGCAGCAAGATC  GAGAGAAATG CTTACACAC  ATGGAGGAGATGATCTTCAAC |

Notes: Hsa, Homo sapiens; Mus, Mus musculus.

**Supplemental References**

1. Yu EZ, Li YY, Liu XH, Kagan E, McCarron RM. Antiapoptotic action of hypoxia-inducible factor-1a in human endothelial cells. *Lab invest* 2004; 84: 553-861.

2. Zhang WH, Tsan R, Huang WC, Wu QY, Chiu CH, Fidler IJ, et al. Survival of Cancer Cells Is Maintained by EGFR Independent of Its Kinase Activity. *Cancer cell* 2008; 13: 385-393.

3. Gao TX, Zheng SY, Li Q, Ran PZ, Sun LJ, Yuan YC et al. Aberrant hypomethylation and overexpression of the eyes absent homologue 2 suppresses tumor cell growth of human lung adenocarcinoma cells. *Oncol Rep* 2015; 34: 2333-2342.

4. Song Y, Wang W, Qu X, Sun S. Effects of hypoxia inducible factor-1alpha (HIF-1alpha) on the growth & adhesion in tongue squamous cell carcinoma cells. *Indian J Med Res* 2009; 129: 154-163.

5. Koivunen P, Lee S, Duncan CG, Lopez G, Lu G, Ramkissoon S et al. Transformation by the R Enantiomer of 2-Hydroxyglutarate Linked to EglN Activation. *Nature* 2013; 483: 484-488.

6. Yuan B, Cheng L, Chiang HC, Xu XJ, Han YJ, Su H et al. A phosphotyrosine switch determines the antitumor activity of ERβ. *J Clin Invest* 2014; 124: 3378-3390.

7. Kim SE, Choi KY. EGF receptor is involved in WNT3a-mediated proliferation and motility of NIH3T3 cells via ERK pathway activation. *Cell Signal* 2007; 19:1554-1564.

8. Wang Y, Liu Y, Malek SN, Zheng P, Liu Y. Targeting HIF1α eliminates cancer stem cells in hematological malignancies. *Cell Stem Cell* 2011; 8: 399-411.
