# Supplementary material for: The EGFR/miR-338-3p/EYA2 axis controls breast tumor growth and lung metastasis
Source: Cell Death Dis. 2017 Jul 13;8(7):e2928–. doi: 10.1038/cddis.2017.325 (PMC5550870; doi:10.1038/cddis.2017.325)
Supplement: Supplementary Table 2 [file cddis2017325x3.doc]

**Supplementary Table S2**. **The real-time PCR primers for miRNA analysis**

| **Gene** | **Forward primer** | **Reverse primer** |
| --- | --- | --- |
| mmu-miR-130b-3p | 5’-GGGCAGTGCAATGATGAAA-3’ | 5’-GTGCAGGGTCCGAGGT-3’ |
| mmu-miR-200a-5p | 5’-GGGCATCTTACCGGACAGTG-3’ | 5’-GTGCAGGGTCCGAGGT-3’ |
| mmu-miR-98-5p | 5’-GGGTGAGGTAGTAAGTTGT-3’ | 5’-GTGCAGGGTCCGAGGT-3’ |
| mmu-miR-27b-5p | 5’-GGGAGAGCTTAGCUGATTG-3’ | 5’-GTGCAGGGTCCGAGGT-3’ |
| mmu-miR-7 | 5’-GGGTGGAAGACTAGTGATTT-3’ | 5’-GTGCAGGGTCCGAGGT-3’ |
| mmu-miR-31-3p | 5’-GGGTGCTATGCCAACATATT-3’ | 5’-GTGCAGGGTCCGAGGT-3’ |
| mmu-miR-222-5p | 5’-GGGTCAGTAGCCAGTGTA-3’ | 5’-GTGCAGGGTCCGAGGT-3’ |
| mmu-miR-26b-5p | 5’-GGGTTCAAGTAATTCAGG-3’ | 5’-GTGCAGGGTCCGAGGT-3’ |
| mmu-miR-338-3p | 5’-GGGTCCAGCATCAGTGATT-3’ | 5’-GTGCAGGGTCCGAGGT-3’ |
| mmu-miR-700-5p | 5’-GGGTAAGGCTCCTTCCTGT-3’ | 5’-GTGCAGGGTCCGAGGT-3’ |
| mmu-miR-669e-5p | 5’-GGGTGTCTTGTGTGTGCAT-3’ | 5’-GTGCAGGGTCCGAGGT-3’ |
| mmu-miR-671-5p | 5’-GGGAGGAAGCCCTGGAGGGG-3’ | 5’-GTGCAGGGTCCGAGGT-3’ |
| mmu-miR-1 | 5’-GGGACATACTTCTTTATATG-3’ | 5’-GTGCAGGGTCCGAGGT-3’ |
| mmu-miR-125a | 5’-GGGTCCCTGAGACCCTTTAAC-3’ | 5’-GTGCAGGGTCCGAGGT-3’ |
| mmu-miR-143-5p | 5’-GGGGGTGCAGTGCTGCAT-3’ | 5’-GTGCAGGGTCCGAGGT-3’ |
| mmu-miR-145-3p | 5’-GGGATTCCTGGAAATACTG-3’ | 5’-GTGCAGGGTCCGAGGT-3’ |
| mmu-U6 snRNA | 5’-GTCTCTTGTGGGCAAGGATG-3’ | 5’-CATGTGAGGGTATTTCTCAG-3’ |
| hsa-miR-338-3p | 5’-GGGTCCAGCATCAGTGATT-3’ | 5’-GTGCAGGGTCCGAGGT-3’ |
| hsa-U6 snRNA | 5’-CTCGCTTCGGCAGCACA-3’ | 5’-AACGCTTCACGAATTTGCGT-3’ |

Notes: mmu, mus musculus; hsa, homo sapiens.
