# Supplementary material for: The EGFR/miR-338-3p/EYA2 axis controls breast tumor growth and lung metastasis
Source: Cell Death Dis. 2017 Jul 13;8(7):e2928–. doi: 10.1038/cddis.2017.325 (PMC5550870; doi:10.1038/cddis.2017.325)
Supplement: Supplementary Figure Legends [file cddis2017325x4.doc]

**Legends for Supplementary Figures**

**Figure S1. EGFR inhibits miR-338-3p expression in 4T1 cells.**

qRT-PCR analysis of miR-338-3p expression in 4T1 cells transfected with increasing amounts of EGFR (A) or in 4T1 cells treated with gefitinib (1 μmol/L) (B) or EGF (100 ng/ml) (C) for the indicated times or in 4T1 cells transfected with EGFR siRNA and treated with EGF (100 ng/ml) (D). Representative immunoblot with the indicated antibodies is shown (A-D). All values shown are mean ± SD of triplicate measurements and have been repeated 3 times with similar results. **P* < 0.05, ***P* < 0.01 versus corresponding control.

**Figure S2. EGFR decreases miR-338-3p expression largely through HIF1α in 4T1 cells.**

(A) Luciferase activity of different miR-338-3p promoter reporters in 4T1 cells transfected with HIF1α or empty vector. Filled circles indicates the position of the putative HIF1α-binding site, and the “X” indicates the mutated HIF1α-binding site. The red letters of each binding region show the putative HIF1α-binding sequences or the mutated HIF1α-binding sequences. (B) Luciferase assay of the indicated miR-338-3p promoter reporters from (A) in 4T1 cells transfected with EGFR and exposed to either normoxic or hypoxic (1% O2) condition. (C) ChIP analysis of HIF1α occupancy on the miR-338-3p promoter or upstream of the promoter in 4T1 cells under normoxic or hypoxic condition. (D) qRT-PCR analysis of miR-338-3p expression in 4T1 cells transfected with EGFR or EGFR plus HIF1α shRNA1 or HIF1α shRNA2 and exposed to either normoxic or hypoxic condition. All values shown are mean ± SD of triplicate measurements and have been repeated 3 times with similar results. **P* < 0.05, ***P* < 0.01 versus corresponding promoter reporter (A, B). ***P* < 0.01 versus corresponding normal IgG (C). **P* < 0.05, ***P* < 0.01 versus corresponding empty vector (D).

**Figure S3. miR-338-3p suppresses EYA2 expression by targeting it’s 3’UTR.**

**(A, B)** Immunoblot analysis of 4T1 cells transfected with miR-338-3p mimics (A) or

anti-miR-338-3p (B). NC, non-specific control for miRNA-338-3p. Scramble,

negative control for anti-miR-338-3p. Histograms under the immunoblots show the

corresponding miR-338-3p mRNA expression levels. (C) miRNA luciferase reporter assays in the indicated breast cancer cells transfected with miR-338-3p and wild-type or mutated EYA2 reporter. Values shown are mean ± SD of triplicate measurements that have been repeated 3 times with similar results. ***P* <0.01 versus corresponding NC or Scramble (A, B). ***P*< 0.01 versus corresponding EYA2 WT (C).

**Figure S4. EGFR increases Eya2 expression in 4T1 cells via HIF1α repression of miR-338-3p.** (A) qRT-PCR and immunoblot analysis of 4T1 cells transfected with empty vector or increasing amounts of EGFR. (B) qRT-PCR and immunoblot analysis of 4T1 cells transfected with EGFR or EGFR plus HIF1α siRNA or EGFR plus anti-miR-338-3p as indicated. (C) qRT-PCR and immunoblot analysis of 4T1 cells transfected with EGFR siRNA or EGFR siRNA plus HIF1α siRNA or EGFR siRNA plus anti-miR-338-3p as indicated. (D) qRT-PCR and Immunoblot analysis of MCF-7 cells transfected with miR-338-3p mimics or anti-miR-338-3p. (E) qRT-PCR and immunoblot analysis of 4T1 cells transfected with HIF1α or HIF1α plus anti-miR-338-3p as indicated. (F) qRT-PCR and immunoblot analysis of 4T1 cells transfected with HIF1α siRNA or HIF1α siRNA plus anti-miR-338-3p as indicated. miR-338-3p expression levels were determined by qRT-PCR (A-E). All values shown are mean ± SD of triplicate measurements and have been repeated 3 times with similar results. **P* < 0.05, ***P* < 0.01 versus corresponding control.

**Figure S5. EGFR regulates cell proliferation via the miR-338-3p/Eya2 axis in 4T1 cells.** (A) 4T1 cells were transfected with EGFR, EGFR plus anti-miR-338-3p or EGFR plus Eya2 siRNA as indicated. Cell proliferation assays were determined by using CCK-8 kit at the indicated times. qRT-PCR shows the expression of miR-338-3p and immunoblot the expression of EGFR and Eya2. (B) 4T1 cells were transfected with EGFR or EGFR plus miR-338-3p as indicated. Cell proliferation was examined as in (A). (C) 4T1 cells were transfected with miR-338-3p or miR-338-3p plus Eya2 as indicated. Cell proliferation was examined as in (A). All values shown are mean ± SD of triplicate measurements and have been repeated 3 times with similar results. **P* < 0.05, ***P* < 0.01 versus corresponding control.

**Figure S6. The miR-338/Eya2 axis regulates cell migration and invasion in 4T1 cells.** (A) Wounding healing assays of 4T-1 cells transfected with miR-338-3p or miR-338-3p plus Eya2. (B) Invasion assays of 4T-1 cells transfected as in (A). (C) qRT-PCR and immunoblot analyses of 4T-1 cells transfected as in (A). qRT-PCR shows miR-338-3p expression. Scale bar, 100 μm (A and B). All values shown are mean ± SD of triplicate measurements and have been repeated 3 times with similar results. ***P* < 0.01 versus corresponding control.

**Figure S7. Validation of the specificity of anti-Eya2, anti-EGFR and the miR-338-3p probe.** (A) The specificity of the anti-Eya2 antibody and the anti-EGFR antibody were determined by preincubation of the antibodies with corresponding purified GST-tagged Eya2 or GST-tagged EGFR or GST alone before immunohistochemical staining. (B) Immunoblot analysis of lysates from MCF7 cells transfected with Eya2 shRNA or EGFR shRNA. (C) Different miR-338-3p expression levels in 3 different breast tissues examined by miRNA in situ hybridization (MISH) (left panel) were confirmed by qRT-PCR (right panel). (D) Positive control (U6) and negative control (Scramble) from the MISH kit were confirmed. Scale bar, 100 μm. All values shown are mean ± SD of triplicate measurements and have been repeated 3 times with similar results. **P* < 0.05, ***P* < 0.01 versus corresponding control.

**Figure S8. A proposed model for EGFR/miR-338-3p/Eya2 axis regulating breast cancer growth and metastasis.** EGFR activates the HIF1α transcription factor and subsequently inhibits miR-338-3p transcription. Decreased miR-338-3p expression increases Eya2 expression by targeting its 3’UTR, thus promoting breast cancer growth and metastasis.
